# Advice alone versus structured detoxification programmes for complicated medication overuse headache (MOH): a prospective, randomized, open-label trial

**DOI:** 10.1186/1129-2377-14-10

**Published:** 2013-02-08

**Authors:** Paolo Rossi, Jessica Veronica Faroni, Cristina Tassorelli, Giuseppe Nappi

**Affiliations:** 1 Headache Clinic, INI Grottaferrata, Via S.Anna s.n.c, 00046, Grottaferrata, Rome, Italy; 2 Headache Science Centre, National Neurological Institute C. Mondino Foundation, Pavia, Italy

**Keywords:** Medication overuse headache, Management, Migraine, Treatment

## Abstract

**Background:**

The aim of this study was to compare the effectiveness of an educational strategy (advice to withdraw the overused medication/s) with that of two structured pharmacological detoxification programmes in patients with complicated medication overuse headache (MOH) plus migraine.

**Methods:**

One hundred and thirty-seven complicated MOH patients participated in the study. MOH was defined as complicated in patients presenting at least one of the following: a) a diagnosis of co-existent and complicating medical illnesses; b) a current diagnosis of mood disorder, anxiety disorder, eating disorder, or substance addiction disorder; c) relapse after previous detoxification treatment; d) social and environmental problems; e) daily use of multiple doses of symptomatic medications. Group A (46 patients) received only intensive advice to withdraw the overused medication/s. Group B (46 patients) underwent a standard detoxification programme as outpatients (advice + steroids + preventive treatment). Group C (45 patients) underwent a standard inpatient withdrawal programme (advice + steroids + fluid replacement and antiemetics preventive treatment). Withdrawal therapy was considered successful if, after two months, the patient had reverted to an intake of NSAIDs lower than 15 days/month or to an intake of other symptomatic medication/s lower than 10 days/month.

**Results:**

Twenty-two patients failed to attend follow-up visits (11 in Group A, 9 in Group B, 2 in Group C, p < 0.03). Overall, we detoxified 70% of the whole cohort, 60.1% of the patients in Group A and in Group B, and 88.8% of those in Group C (p < 0.01).

**Conclusions:**

Inpatient withdrawal is significantly more effective than advice alone or an outpatient strategy in complicated MOH patients.

## Background

Medication overuse headache (MOH) is a relevant public health problem worldwide [1,2]. Population-based studies in several European countries have reported prevalence rates of MOH ranging from 0.9 to 1.8%, making it the third most frequent headache disorder after tension-type headache and migraine [3-7].

Medication overuse headache has detrimental effects on patients’ quality of life and it places a high economic burden on society [1,2].The recently published findings of the Eurolight project show that the mean per-person annual costs of MOH are 3 times higher than those of migraine and 10 times higher than those of tension-type headache [8].

The diagnosis of MOH has been greatly facilitated by the *new appendix criteria for a broader concept of chronic migraine*, published in 2006 by the IHS [9]. These simplified criteria, which eliminate the need for headache resolution or reversion to the previous episodic pattern after drug discontinuation in order to confirm the diagnosis, are helping physicians to recognise MOH and thus to remove the diagnostic barriers to proper care of affected patients.

Drug withdrawal is considered the treatment of choice for MOH, even though this view has recently been challenged [1,2,10-16]. Drug withdrawal is however approached and performed very differently within and across countries. Furthermore therapeutic recommendations for the acute phase of detoxification vary considerably between studies [1,2,17-19]. Other reasons for the heterogeneity in MOH management are the shortage of controlled treatment studies, meaning that treatment recommendations are based mainly on expert opinions rather than on solid scientific evidence [20], and the fact that MOH is, in many respects, a heterogeneous disorder. MOH group indeed encompasses a spectrum of conditions that vary in terms of phenotypical characteristics, type and amount of drugs overused, comorbidities and response to prophylactic treaments. In order to improve the management of MOH, some authors have pragmatically suggested to subdivide MOH into two clinical subtypes: simple and complex [21-23]. In accordance with this approach we previously demonstrated that in MOH patients with low medical needs and migraine as the primary headache type advice and education were as effective as structured inpatient and outpatient detoxification programmes in achieving withdrawal of the overused medication/s [24]. More recently, other groups studying “mildly” affected MOH populations have confirmed the effectiveness of brief educational intervention alone in reducing medication overuse and headache chronification and also the similar efficacy between inpatient and outpatient programmes [25-27]. These studies thus lend support to the recommendation that uncomplicated MOH should, in the first instance, be tackled through outpatient withdrawal programmes [14,20,26-28]. Conversely, complicated forms, such as those with opioids, barbiturates or benzodiazepines overuse, or presenting psychological problems or medical comorbidities that could impact on outpatient withdrawal, are generally considered for inpatients withdrawal, as are those who have previously failed outpatient detoxification programmes or who lack the necessary motivation [14,17,18,27]. In partial support of the simple-vs-complex approach, we showed in a previous study that educational intervention alone is significantly more effective in patients with simple MOH as opposed to patients with complicated MOH (92.1% vs 65.3% of responders, respectively); although the level of adherence to treatment did not differ between the two groups [24] and notwithstanding the fact that a success rate of 65.3% was obtained in complicated MOH.

The aim of this study was to compare the effectiveness of a simple withdrawal strategy, based exclusively on intensive advice to withdraw the overused medication/s, with that of two structured pharmacological detoxification programmes in a cohort of patients with complicated MOH in whom migraine was the primary headache type.

## Methods

For the purpose of this study, complicated MOH was defined by the presence of at least one of the following conditions [29]: a) comorbidity with other clinically relevant painful condition (e.g. chronic painful disorders such as fibromyalgia or low back pain, neuropathic pain, etc.); b) ongoing or recent comorbidity with psychiatric disorders (i.e. mood disorder, anxiety disorder, substance addiction disorder or eating disorder), as assessed by the Structured Clinical Interview for DSM-IV Axis I Disorders (SCID-I), Clinician Version [30]; c) moderate/severe psychosocial and environmental problems as defined by DSM-IV Axis IV, assessed at the end of the initial visit by means of a structured interview; d) daily, or almost daily, use of multiple doses of symptomatic medication/s (more than 3 doses/day of analgesics or more than 2 doses/day of triptans, ergots, combinations of acute medications, or analgesics in combination) or anticipatory use of symptomatic medication/s; e) relapse into overuse following previous detoxification treatment.

This set of criteria for defining complicated MOH was deemed to represent the best combination of existing criteria (already proposed in the literature) and the authors’ personal experience in managing MOH patients attending headache centres in Italy, and it has already proved to be highly sensitive in identifying MOH patients in whom effective drug withdrawal may be obtained through the imparting of advice alone [29].

It has been established that abrupt discontinuation of an overused medication may lead to serious withdrawal symptoms such as hypotension, tachycardia, vomiting, intense nervousness, sleep disturbances and even hallucinations and seizures, particularly in patients overusing opioids, barbiturates and tranquillisers, and that these symptoms are best treated under medical supervision in patients with significant complicating medical conditions [1,14,19,20]. Therefore, for ethical reasons, in this study we excluded patients with severe major depression and/or severe psychiatric illnesses and those likely to encounter problems as withdrawal treatment outpatients. Thus, the final set of exclusion criteria were: a) co-existent severe medical (e.g. uncontrolled arterial hypertension, uncontrolled diabetes mellitus, ischaemic cardiopathy, etc.) or psychiatric illness; b) overuse of opioids and/or barbiturate-containing agents; c) treatment with migraine prophylactic drugs within the past three months; d) pregnancy or breastfeeding.

Consecutive new patients, aged 16–65 years, affected by complicated MOH [29] in whom the primary headache was migraine attending a specialist headache centre (INI Grottaferrata Headache Clinic) were evaluated over an eighteen-month period (July 2009 to December 2010).

**Figure 1:**
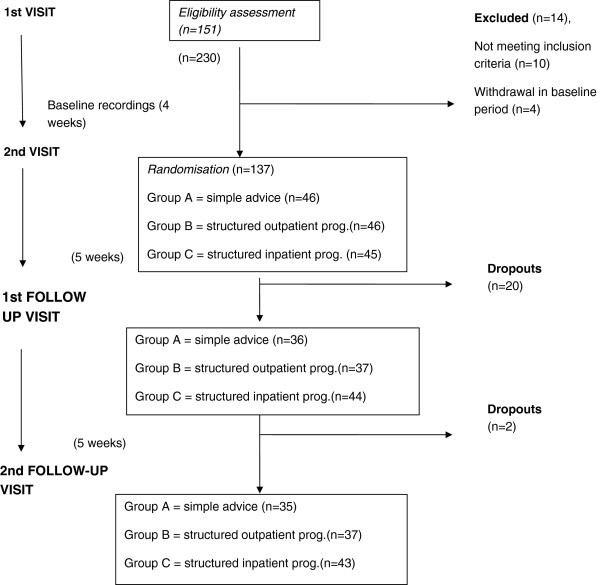
Outline of the trial.

The patients were evaluated prospectively and the study lasted 14 weeks for each subject (Figure 1). After the first visit, eligible subjects were asked to keep a diagnostic headache diary for four weeks, simply recording their headache pattern and drug use (baseline period). This period was purely observational and at this time no diagnosis or therapeutic indications were given. At the second visit, at the end of this baseline period, the patients still fulfilling the inclusion criteria were randomly assigned (using a computer-generated random number sequence), in equal numbers, to three different treatment groups [24]. Group A received only advice to withdraw the overused medication/s; Group B underwent a standard outpatient detoxification programme consisting of: 1) advice to abruptly withdraw the overused medication/s, 2) prednisone p.o. during the first 10 days (60 mg/day, 2 days; 40 mg/day, 2 days; 20 mg/day, 6 days) [24], 3) individualised preventive treatment begun on day 1 (the preventive agent was chosen on the basis of side-effect profile, comorbid conditions, the patient’s needs and preferences, and the patient’s previous therapeutic experiences). Group C underwent a standard inpatient detoxification programme. After receiving advice about the need to withdraw symptomatic medications, patients in this group were admitted as inpatients to the INI Grottaferrata Headache Clinic for a 10-day period during which they received: 1) abrupt discontinuation of the overused medication/s, 2) close observation and support for 8–10 days, 3) prednisone p.o. (60 mg/day, 2 days; 40 mg/day, 2 days; 20 mg/day, 6 days), 4) individualised preventive treatment as from day 1, and 5) parenteral fluid replacement and administration of antiemetics (metoclopramide i.v).

In order to standardise the educational part of the treatment programme, all advice was issued by the same physician (PR). The advice to discontinue the overused medication/s was given verbally [24] and its imparting, which took about 15 minutes during the second consultation, was structured as follows: a) the role of medication overuse in making headache chronic and in reducing the effectiveness of preventive and behavioural treatments was explained; b) the phenomenon and symptoms of withdrawal headache were explained in detail; c) the beneficial long-term effects, on migraine natural history, of reducing symptomatic medication intake (which include reduction of the migraine-reinforcing properties of short-term pain relief) were emphasised; d) anticipatory use of medication was discouraged; and e) the superiority of the detoxification programme over other therapeutic options was emphasised. In patients assigned to Group A the physician emphasised the importance, for patients, of playing an active role in the management of their headache, without relying solely on medication.

For the treatment of withdrawal headache and associated symptoms, patients could be prescribed the following acute drugs, according to their personal medical history (the rationale was that patients should not use the drug that she or he had previously been abusing) and headache characteristics:

a) Antiemetics (metoclopramide 10 mg i.m. or p.o.1 to 3 times per day; chlorpromazine, 25–50 mg i.m. or p.o., domperidone 30 mg rectally or 10 mg p.o.), and (with intake limited to no more than two days per week)

b) Acetaminophen (1000 mg p.o. or rectally or i.v on demand, maximum dosage 3 g × day) or naproxen (500 mg p.o. or p.r., maximum dosage 1000 mg × day) or indomethacin (100 mg p.o. or p.r., or 50 mg i.m., maximum dosage 200 mg × day)

c) Eletriptan 40 mg p.o., frovatriptan 2.5 mg p.o., almotriptan 12.5 mg p.o., or rizatriptan 10 mg p.o.

Furthermore, the patients were asked to fill in a detailed diagnostic headache diary. The patients assigned to outpatient treatment groups were also told that they could contact the headache centre for medical help and support, should this be needed.

Follow-up visits were scheduled during the 5th and the 10th week after the start of the detoxification programme. All patients failing to attend the follow-up visits were called by telephone within three months from the start of the detoxification programme in order to ascertain their reasons for not adhering to the treatment programme. As in our previous studies [24,29], the patients were not informed that they were included in a study assessing the effectiveness of simple advice as a withdrawal strategy for MOH. This approach was authorised by the ethics committee of our institute. Therefore, no signed, informed consent was obtained. The research was in compliance with the Helsinki Declaration.

### Outcome measures

The primary outcome measures were: a) responders (number of), defined as those subjects who, two months after the start of withdrawal of the overused drug/s, had reverted to an intake of NSAIDs lower than 15 days/month or to an intake of other symptomatic medication/s lower than 10 days/month; b) adherence to the treatment, expressed as the number of patients who completed the follow-up visits; c) responders with headache improvement (number of), defined as those patients who, two months after the start of the drug withdrawal, experienced more than 50% reduction in headache frequency from baseline.

As secondary outcome measures we considered the percentage reduction, two months after the start of the withdrawal, in the number of headache days/month, the number of days with use of symptomatic medication/month, and the number of symptomatic medications/month.

### Power calculation and statistical analysis

The main target parameter was the number of responders. On the basis of the results of previous studies, we assumed a responder rate of 55% in the no-intervention group and of 90% in the structured intervention groups, i.e. the study had to have the power to detect a difference of 35%. In applying the above-mentioned percentages, using a power of 0.8% and a significance level of 5%, a minimum of 35 patients per treatment group was required (Fisher’s exact test, two-tailed). We thus needed a total of 105 patients to complete the study. Given the impossibility of performing a satisfactory *a priori* analysis of the other outcome measures, the adherence to treatment criterion and the other target criteria were subjected to exploratory statistical analysis.

Multi-group comparisons were performed using the one-way analysis of variance for continuous variables and the non-parametric Kruskal-Wallis test for non-normally distributed data. The chi-square test and Fisher’s exact test with Freeman-Halton extension (when applicable) were used for multi-group comparisons of categorical variables. Given that we were performing multiple testing, the level of significance was set at p < 0.025. The analysis was carried out using SPSS 11 for Windows (SPSS Inc., Chicago, IL).

## Results

One hundred and fifty-one patients were evaluated during the study period; 10 were excluded because they did not meet the inclusion criteria (four of these overused butalbital containing agents, no one overuse opioids). Of the remaining 141 patients, included in the study, four withdrew during the baseline period. Table 1 presents the demographic and clinical characteristics of the patients randomised to the three different detoxification groups. Of the 137 patients forming the final sample, 110 were females (80.3%) and 27 (19.7%) were males, mean age 46±12 years. Forty-eight patients (35%) overused NSAIDs, 31 (22.7%) overused NSAIDs in combination (in all cases indomethacin plus caffeine plus prochlorperazine), three (2.2%) overused ergots, 37 overused triptans (27%), and 18 (13.1%) overused combinations of acute medications (14 used triptans + NSAIDs, 3 used NSAIDs in combination + triptans, 1 used triptans + ergotamine). Sociodemographic variables, migraine subtype, migraine duration, MOH duration, number of headache days per month, and number and type of overused medication(s) did not differ significantly between the three groups (Table 1). Table 2 shows the prevalence of the different factors complicating MOH in the three groups of patients.

**Table 1:** Demographics and headache characteristics of the study population

	***Group A (n = 46)***	***Group B (n = 46)***	***Group C (n = 45)***	***Statistics***
**Sex** n° (%)				NS§
F	39	37	34	
M	7	9	11	
**Age** mean ± SD (yrs)	44.9 ± 11	46.2 ± 12	46.3 ± 11.4	NS¤
(median)	(48)	(48)	(49)	
**Educational level** n° (%)				
Secondary school or above	30	31	27	NS§
Primary or middle school	16	15	18	
**Employed** n°(%)				
Yes	29	28	25	NS§
No	17	18	20	
**Marital status** n° (%)				
Single	8	7	5	NS§
Married	32	32	35	
Widowed/divorced	6	7	5	
**Migraine subtype** n° (%)				
With aura	5	3	5	NS*
Without aura	41	43	40	
**Duration of migraine**				
mean±SD (yrs)	25.8 ± 12.8	26.0 ± 12	26.1 ± 11.7	NS¤
(median)	(29)	(29)	(30)	
**Duration of MOH**				
mean±SD (yrs)	3.3 ±3.3	2.9 ± 3.1	3.4 ± 2.9	NS¤
	(3)	(2)	(3)	
**Number of headache days/month**				
mean±SD	24.9 ± 6	25.4 ± 6.7	25.4 ± 6.4	NS¤
(median)	(30)	(30)	(30)	
**Number of days with use of symptomatic medication/month**				
mean±SD	23.9 ± 5.4	24.1 ± 4.8	24.3 ± 4.9	NS¤
(median)	(30)	(30)	(30)	
**Overused drugs** n (%):				NS*
· Analgesics	16	14	18	
· Ergotamine	1	1	1	
· NSAIDs in combination	10	11	10	
· Combination of acute medications	6	6	6	
· Triptans	13	14	10	
**Number of overused medications/month**				NS¤
mean ±SD	37.2 ± 28.4	36.9 ± 32.4	38.2 ± 31	
(median)	(38)	(38)	(40)	

NS = not significant, *Fisher’s test, § Chi-square test, ¤ Kruskal-Wallis test.

**Table 2:** Prevalence of clinical factors defining MOH as complicated

	**Group A (n = 46)**	**Group B (n = 46)**	**Group C (n = 45)**	**Statistics**
**Psychiatric comorbidity n° (%)**	26 (56.5%)	26 (56.5%)	30 (66.6%)	NS*
· Anxiety disorder	16 (34.7%)	17 (36.9%)	19 (42.2%)	
· Mood disorder	21 (45.6%)	21 (45.6%)	22 (48.8%)	
· Anxiety and mood disorder	10 (21.7%)	10 (21.7%)	11 (24.4%)	
· Eating disorder	3 (6.5%)	2 (4.3%)	3 (6.6%)	
*Psychiatric comorbidity alone*	8 (17.4%)	8 (17.4%)	11(24.4%)	
**Psychosocial and environmental problems n° (%)**	16 (34.7%)	10 (21.7%)	12 (26.6%)	NS*
*Psycho-social and environmental problems alone*	6 (13%)	4 (8.7%)	4(8.8%)	
**Relapsers n° (%)**	6 (13%)	9 (19.5%)	8 (17.7%)	NS§
**Daily use of multiple drugs n° (%)**	11 (23%)	17 (36.9%)	17 (37.7%)	NS§
Medical illnesses n° (%)**	4 (8.7%)	2 (4.3%)	2 (4.4%)	NS*

*Fisher’s test, § Chi-square test.

**chronic low back pain = 2, fibromyalgia =2, recent intervention for colon cancer = 1, severe obesity = 2, chronic C hepatitis on antiretroviral therapy = 1, NS = not significant.

In group B, 16 patients received valproic acid (500–1000 mg/day) as preventive medication, 11 received beta-blockers (metoprolol 100–200 mg/ day), nine received amitriptyline (25–75 mg/day), and 10 received topiramate (75 mg/day). In group C, 17 patients received valproic acid (500–1000 mg/ day) as preventive medication, eight received beta-blockers (metoprolol 75–100 mg/ day), eight received amitriptyline (30–60 mg/ day) and 12 received topiramate (50–75 mg/ day).

Twenty-two patients (16%) dropped out of the study (11 in Group A, 9 in Group B and 2 in Group C, p < 0.025, Table 3). The reasons given for missing follow-up visits were: lack of time (two in Group A, two in Group B, all at the first follow-up), lack of motivation (two in Group A, one in Group B, one in Group C, all at the first follow-up), the decision to seek other medical help (three in Group A, one in Group B, both at the first follow-up), fear of side effects (one in Group A, two in Group B, both at the first follow-up), health problems (one in Group A, one in group C, both at the second follow-up) and worsening of headache (two in Group A, three in Group B, both at the first follow-up). No differences in headache characteristics or demographics were found between the patients from the three groups completing and not completing the follow-up visits (all, p > 0.025).

**Table 3:** Primary and secondary outcome measures

	***Group A (n = 46)***	***Group B (n = 46)***	***Group C (n = 45)***	***Statistics***
**Patients missing follow-up visits** n(%)	11 (23.9)	9 (19.5)	2 (4.4)	p < 0.025*
**Responders** n(%)	28 (60.8)	28 (60.8)	40 (88.9)	p = 0.003*
**Responders with headache improvement** n (%)	25 (54.3)	26 (56.5)	38 (84.4)	p = 0.003§
**Percent reduction in number of headache days/month** mean ± SD (median)	44 ± 25 (50)	49.8 ± 28 (52)	73 ± 22 (76)	p < 0.001¤
**Percent reduction in the number of days with use of symptomatic medication** mean ± SD (median)	62.5 ± 23 (64)	63.6 ± 26 (64)	75.2 ± 23 (78)	p = 0.001¤
**Percent reduction in the number of symptomatic medication** mean ± SD (median)	67.8 ± 18 (68)	69.7 ± 22 (70)	83.3 ± 20 (84)	p = 0.001

*Fisher’s test, § Chi-square test, ¤ Kruskal-Wallis test.

After two months, 115 patients had completed the follow-up visits (35 in Group A, 37 in Group B, 43 in Group C). Of these, 96 (84.2%) were considered responders (28 in Group A, 28 in Group B, 40 in Group C, p = 0.003, Table 3). Of all the patients included in the study we successfully detoxified 70% (60.8% in Groups A and B, and 88.9% in Group C, p = 0.003 Table 3). At two months, 25 patients in Group A (56.5%), 26 in Group B (56%) and 38 in Group C (84.4%) recorded a more than 50% reduction in headache frequency from baseline (p = 0.003) and were thus considered responders with headache improvement.

In the patients completing the study, the percent reduction in the number of headache days/month was 44 ± 25 in Group A, 49.8 ± 28 in Group B and 73 ± 22 in Group C (p < 0.001). The percent reduction in the number of days with use of symptomatic medication was 62.5 ± 23 in Group A, 63.6 ± 26 in Group B, and 75.2 ± 23 in Group C (p < 0.001). The percent reduction in the number of symptomatic medications/month was 68,1 ± 18 in Group A, 69.7 ± 22 in Group B and 84.3 ± 20 in Group C (p < 0.001)

## Discussion

The main finding of this study was that a structured inpatient detoxification programme was more effective, in the short-term, than advice alone or than a structured outpatient programme in achieving withdrawal of the overused medication(s) in patients with complicated MOH who had migraine as their primary headache type. In addition, contrary to the pattern emerging in simple MOH [24], outpatient strategies were associated with a lower level of adherence to treatment: 21.8% of the patients allocated to outpatient strategies did not complete the study, whereas only 4.6% of the patients allocated to the inpatient programme dropped out of the study.

As in previous studies, conducted in patients with simple or mild MOH [14,24,25], early administration of a preventive treatment in an outpatient setting did not improve the effectiveness of the withdrawal treatment when compared with simple advice to withdraw the overused drug(s). This suggests that hospitalisation was the factor that, in our complicated MOH patients, made the difference versus the other detoxification strategies in both responders and adherence to treatment measures. This is probably because the inpatient approach allowed a more strict control of medication intake, the prompt and intensive treatment of withdrawal symptoms, a better patient support, and isolation of patients from the socio-environmental pressures liable to reinforce drug overuse behaviour.

In the recently published EFNS headache panel guideline on the treatment of MOH [20], the authors concluded that “the type of withdrawal strategy (inpatient, outpatient, advice alone) does not influence the success of the treatment and the relapse rate in general” (level A recommendation). Two more recent studies, conducted in Europe, seem to support this statement [27,28]. Creac’h et al. compared the efficacy of inpatient and outpatient withdrawal programmes in 82 consecutive patients with MOH in an open-label prospective randomised trial. The finding that hospitalisation did not improve either the short or the long-term prognosis of these patients prompted the authors to recommend outpatient withdrawal in the first instance in patients with MOH [27]. Munksgaard et al. investigated the effectiveness of two structured outpatient detoxification programmes in 98 treatment-resistant patients with MOH, i.e. patients previously unsuccessfully treated by specialists [28]. Both programmes proved to be highly effective in the short and the long term, which suggests that most MOH patients, even those regarded as treatment-resistant, may be cured in an outpatient setting. However, these two studies [27,28], like all the previous research comparing different approaches to withdrawal therapy [14,20], did not include patients with complicated MOH as defined herein. The present study, in which hospitalisation was found to be superior to outpatient strategies, is thus the first to investigate the efficacy of different withdrawal strategies in complicated MOH. Nevertheless, it is worth noting that, as in our previous study [29], simple educational intervention alone proved sufficient to detoxify the majority (60%) of the complicated MOH patients and 80% of the patients completing the study. These data suggest that compliance with outpatient strategies is the main problem in complicated MOH patients, given that almost 20% of them were somewhat dissatisfied by an approach based essentially on the imparting of advice to withdraw the symptomatic drug/s. A recent study showed that MOH patients request information about the disease and want to know how they might actively participate in the treatment of it [31]. It is possible, therefore, that compliance might be improved by taking patient preferences into account when choosing the withdrawal programme [27]. In view of hospitalisation costs, particularly in the current economic climate, our experience with complicated MOH patients [29] suggests that advice to withdraw the overused medication/s, on the basis of its effectiveness, safety and cost [14], should be the first step in a step-care approach to MOH management that takes into account patients’ preferences. In case of failure, inpatient detoxification should be offered in order to grant a higher rate of success. This approach seems the most adequate one based on the present and on published data, at least until convincing evidence is provided in favour of a stratified approach in which patients are assigned to different treatments according to the extent of their medical needs.

Several possible limitations of our study preclude the drawing of definite conclusions regarding the use of simple advice as a withdrawal strategy for MOH. The headache clinic setting may have favoured the productive doctor-patient alliance that is essential for successful educational interventions. Thus, the reproducibility of our findings in different settings, such as primary care, remains uncertain.

Furthermore, the classification of MOH patients into simple and complicated subtypes is not immediate and requires the administration of time-consuming structured interviews by experienced physicians. Recent work in Norway with the Severity of Dependence Scale (SDS) suggests that this simple and validated questionnaire is useful for predicting the outcome of MOH after an educational intervention in the general population [32]. Therefore, SDS might be a helpful tool for identifying MOH patients with higher medical needs.

Third, this study included only MOH patients who had migraine as their primary headache; these patients have a better prognosis than those with other headache types, such as tension-type headache [1,14].

Finally, the proposed criteria for medical comorbidity (the presence of coexistent significant and complicating medical illnesses) might benefit from a more accurate definition deriving from reproducibility study involving different physicians.

## Conclusions

Inpatient withdrawal is significantly more effective than advice alone or an oupatient strategy in complicated MOH patients.

## Competing interest

The authors declare that they have no competing interests.

## Authors’ contribution

PR had the original idea for the study and together with JVF, CT and GN planned the overall design. PR prepared the initial draft and was the main author of the present manuscript. PR and JVR carried out the study. CT supported in the design of the study and with scientific input regarding headache. All authors have read, revised and approved the final manuscript.
